# Telomere length measurement by qPCR in birds is affected by storage method of blood samples

**DOI:** 10.1007/s00442-017-3887-3

**Published:** 2017-05-25

**Authors:** Sophie Reichert, Hannah Froy, Winnie Boner, Theresa M. Burg, Francis Daunt, Robert Gillespie, Kate Griffiths, Sue Lewis, Richard A. Phillips, Dan H. Nussey, Pat Monaghan

**Affiliations:** 1 Department of Animal and Plant Sciences, University of Sheffield, Sheffield, UK; 2 Institute of Biodiversity, Animal Health & Comparative Medicine, University of Glasgow, Glasgow, UK; 3 Institute of Evolutionary Biology, University of Edinburgh, Edinburgh, UK; 4 British Antarctic Survey, High Cross, Cambridge, UK; 5 Department of Biology, University of Lethbridge, Lethbridge, Canada; 6 Centre for Ecology and Hydrology, Edinburgh, UK

**Keywords:** Wandering albatross, Zebra finch, FTA cards, DNA, Telomeres, Ageing, Long-term field study

## Abstract

Given the potential role of telomeres as biomarkers of individual health and ageing, there is an increasing interest in studying telomere dynamics in a wider range of taxa in the fields of ecology and evolutionary biology. Measuring telomere length across the lifespan in wild animal systems is essential for testing these hypotheses, and may be aided by archived blood samples collected as part of longitudinal field studies. However, sample collection, storage, and DNA extraction methods may influence telomere length measurement, and it may, therefore, be difficult to balance consistency in sampling protocol with making the most of available samples. We used two complementary approaches to examine the impacts of sample storage method on measurements of relative telomere length (RTL) by qPCR, particularly focusing on FTA (Flinders Technology Associates) cards as a long-term storage solution. We used blood samples from wandering albatrosses collected over 14 years and stored in three different ways (*n* = 179), and also blood samples from captive zebra finches (*n* = 30) that were each stored using three different methods. Sample storage method influenced RTL in both studies, and samples on FTA cards had significantly shorter RTL measurements. There was no significant correlation between RTL measured in zebra finch blood on FTA cards and the same samples stored either as frozen whole blood or as extracted DNA. These results highlight the importance of consistency of sampling protocol, particularly in the context of long-term field studies, and suggest that FTA cards should not be used as a long-term storage solution to measure RTL without validation.

**Electronic supplementary material:**

The online version of this article (doi:10.1007/s00442-017-3887-3) contains supplementary material, which is available to authorized users.

## Introduction

Telomeres are highly conserved, non-coding, repetitive DNA sequences that cap the ends of eukaryotic chromosomes. Their main function is to protect coding DNA from loss during cell division, and to prevent chromosome ends from being misidentified as double-stranded breaks by DNA repair machinery (Blackburn 1991). Telomeric DNA is progressively lost because of the inability of linear chromosomes to be completely replicated by DNA polymerase (Blackburn 1991), and this loss can be further aggravated by oxidative stress (von Zglinicki 2002). In the absence of telomere restoration, telomeres shorten with each cell division. Once telomeres reach a critical length, cell division arrest or cell senescence occurs (Harley et al. 1990; Blackburn 1991; Armanios and Blackburn 2012). Telomeres, therefore, play a crucial role in the maintenance of genomic integrity and in the replicative potential of cells (Blackburn 2000).

Until recently, most studies in the field of telomere biology have been conducted using cultured cells and model organisms in the framework of biomedical research focusing on the role of telomere dynamics in human ageing and disease (Aubert and Lansdorp 2008). However, there is increasing appreciation of the potential for telomeres to link lifestyle with health (Monaghan and Haussmann 2006; Monaghan 2010; Lin et al. 2012), survival, or lifespan in a wider range of organisms (Cawthon 2002; Bize et al. 2009; Heidinger et al. 2012). Many evolutionary biologists and ecologists are now intrigued by the role telomere dynamics may play in the evolution of life histories (Monaghan and Haussmann 2006; Monaghan 2010; Stier et al. 2015). Over the past few years, this has led to an increase in the number of studies in the fields of ecology, physiology, and evolutionary biology exploring telomere dynamics in a wider variety of taxa, in correlative and experimental studies in both the field and the laboratory (Nussey et al. 2014).

Given this increase in telomere studies, recent papers have raised important concerns regarding the methods used to assess telomere length (Horn et al. 2010; Haussmann et al. 2011; Nussey et al. 2014). The way in which samples are collected and stored, and the DNA extraction protocol, will influence DNA quality and quantity (Nakagawa et al. 2004; Haussmann et al. 2011; Smith et al. 2011; Nussey et al. 2014), which will, in turn, influence both the method chosen to measure telomere length, and the results of the assay (Cunningham et al. 2013; Nussey et al. 2014). Until now, studies and reviews have mainly focused on comparing methods for DNA extraction (Cunningham et al. 2013; Denham et al. 2014; Hofmann et al. Hofmann et al. 2014; Raschenberger et al. 2016; Seeker et al. 2016) and telomere measurement (Horn et al. 2010; Aviv et al. 2011; Olsen et al. 2012; Martin-Ruiz et al. 2014; Montpetit et al. 2014; Nussey et al. 2014). Recently, Zanet et al. (2013) found that telomere length measurement by qPCR may vary depending on sample storage method, though Tolios et al. (2015) found a minimal effect of sample storage on relative telomere length. However, there are still relatively few studies that have explored the impact of sample storage conditions on telomere length measurement.

An increasing number of ecological and evolutionary biology studies on telomeres are conducted on wild animals, but seldom in the context of long-term field studies (Bize et al. 2009; Pauliny et al. 2012). Considerations about methodological consistency are particularly important in these circumstances, since samples collected as time series are rare and very valuable, and are often archived over a period of years. This can be especially useful when the species is long-lived, since it allows individuals to be sampled across their lifetimes. Measuring telomere length in such longitudinal samples is a very appealing prospect, since it provides insights into telomere dynamics within individuals, which is extremely unusual in wild systems (Bize et al. 2009; Pauliny et al. 2012). However, given the rapid advance in technological methods, and the inevitable changes in the products and protocols available for processes like DNA extraction and sample storage over time, it may be difficult to balance consistency in sampling protocol with making the most of available samples.

One possibility for sample storage is to store dried blood samples on Whatman Flinders Technology Associates (FTA) cards. These cards are impregnated with chemicals that lyse cells, denature proteins, and protect nucleic acids from nucleases, oxidation, and UV damage, making them suitable for long-term storage at room temperature and subsequent qPCR analysis (Pezzoli et al. 2007). This storage technique has been used largely for biomedical assays for disease diagnosis, but also to measure immunological, metabolomic, and toxicokinetic parameters (Sharma et al. 2014). In the context of ecological and evolutionary biology research, the FTA card can prove convenient for field studies as freezing is not required. Although FTA cards are considered a suitable storage method for blood samples prior to qPCR analysis, little is known about the suitability of this storage method specifically for measuring telomere length. To our knowledge, only one study of humans (Zanet et al. 2013) has investigated this issue, finding that telomere length measurements by qPCR were longer in samples stored on FTA cards for 9 days compared to those measured in whole blood, though the correlation was high. However, results may vary depending on the model organism; in particular, since red blood cells are nucleated in birds, findings might differ in avian samples.

The aim of this paper is to assess whether telomere length measured by qPCR can be affected if samples are stored on FTA cards, and more generally to evaluate the effect of storage method on telomere length measurement. We originally aimed to investigate telomere dynamics in a long-lived seabird, the wandering albatross (*Diomedea exulans*), for which we only had access to archived samples stored using a number of common storage methods. The results suggested a profound influence of storage method on telomere length measurement, but the longitudinal nature of the study meant that sample storage method was confounded with other variables such as storage duration. We followed up with an experimental comparison in captive zebra finches (*Taeniopygia guttata*), which was designed to specifically test for impacts of different storage methods in a controlled manner. We, therefore, use two complimentary approaches, involving measurement of telomere length in samples from: (i) wandering albatrosses from a long-term field study (blood samples taken longitudinally over a period of 14 years, and stored in different ways), and (ii) adult zebra finches in captivity (the same blood samples from 30 individuals stored in different ways).

## Methods

### Sample collection, storage, and DNA extraction

#### Albatross study

Blood samples were collected at a wandering albatross breeding colony on Bird Island, South Georgia (54°00′S, 38°03′W), which has been studied since the late 1950s (Croxall et al. 1990; Froy et al. 2013). Birds of known age (4–40 years) were blood sampled during the incubation period of the 1998 (*n* = 16), 1999 (*n* = 2), 2000 (*n* = 69), and 2012 (*n* = 124) breeding seasons. Blood samples from different years were processed and stored in different ways, because they were collected for different purposes (Table 1a). All samples were taken by superficial venipuncture of the tarsal vein.

**Table 1:** Different sample collection, processing, storage, and DNA extraction methods used for A wandering albatross blood samples collected at Bird Island, South Georgia, B zebra finch blood samples, which were used to measure relative telomere length

Year	*n*	Blood sampling	Long-term storage			Extraction	
			State	Conditions	Duration	Timing	Method
(a)							
1998	13	Needle and syringe	Extracted DNA in Tris_EDTA low buffer	Frozen at −20 and −80 °C	14–15 years	Prior to long-term storage	Modified Chelex protocol
1999	2						
2000	56	Needle and syringe	Red blood cells in Tris–EDTA-SDS buffer	Frozen at −2 and −80 °C	13 years	Prior to qPCR	Nucleospin^®^ Blood Kit
2012	108	Pinprick with needle	Whole blood on FTA^®^ClassicCards	Separate ziplock bags at room temp	1 year	Prior to qPCR	Nucleospin^®^ Tissue Kit

Year	*n*	Blood sampling	Long-term storage			Extraction	
			State	Conditions	Duration	Timing	Method
(b)							
2014	30	Needle and capillary	Extracted DNA	Frozen at −80 °C	2 months	Prior to long-term storage	Nucleospin^®^ Blood QuickPure Kit
2014	30	Needle and capillary	Whole blood	Frozen at −80 °C	2 months	Prior to qPCR	Nucleospin^®^ Blood QuickPure Kit
2014	30	Needle and capillary	Whole blood on FTA^®^ClassicCards	Storage at room temp	2 months	Prior to qPCR	Nucleospin^®^ Blood QuickPure Kit

The samples collected in 1998 and 1999 were initially stored as whole blood in ethanol. Genomic DNA was extracted approximately 3 months after sample collection using a modified Chelex protocol, adapted from Walsh et al. (1991) as described in Burg and Croxall (2001). The extracted DNA was subsequently stored in a Tris–EDTA low buffer (1XTE) at −20 or −80 °C. Samples collected in 2000 were separated into plasma and red and white cells by centrifugation. The red blood cell fraction used in this analysis was stored in a Tris–EDTA-SDS buffer at −20 or −80 °C until DNA extraction. Prior to telomere measurement, DNA was extracted using the Macherey–Nagel Nucleospin^®^ Blood Kit (Macherey–Nagel, Düren, Germany) by resuspending 3–4 μl of red blood cells in 196 μl of PBS and following the manufacturer’s protocol. Samples collected in 2012 were stored on FTA^®^ClassicCards manufactured by Whatman^®^BioScience. Genomic DNA was extracted from the FTA^®^ cards using a Macherey–Nagel Nucleospin^®^ Tissue Kit (Macherey–Nagel, Düren Germany) by adding 15–30 mm^2^ of blood-covered FTA cards to a 1.5 ml micro centrifuge tube, omitting the 10 min incubation at 94 °C and following the manufacturer’s support protocol for dried blood spots, eluting DNA in 70 μl of elution buffer BE. DNA integrity was verified by gel electrophoresis for 18 samples. All samples were tested for DNA concentration and purity using a NanoDrop 8000 Spectrophotometer (ThermoScientific). Twenty-five samples with 260/280 <1.7 or >2.6, or 260/230 ratio <1.6 or >2.8 were excluded (leaving the final sample sizes shown in Table 1a).

#### Zebra finch study

Blood samples were collected from the brachial vein of 30 captive adult zebra finches (100–150 µL from 16 males and 14 females aged 1–8 years)—collection year: 2014. The samples were divided into three sets and stored in three different ways for 2 months: the first set of samples were kept on ice and gDNA extracted immediately, with long-term storage as extracted DNA at −80 °C; the second set were frozen as whole blood and stored at −80 °C prior to analysis; and the final set were stored at room temperature on FTA cards (FTA^®^ClassicCards manufactured by Whatman^®^BioScience) as recommended by the manufacturer. DNA extractions were performed using Nucleospin^®^ Blood QuickPure kit (Macherey–Nagel, Düren Germany) (Table 1b). Quantity and purity of extracted DNA were verified with a NanoDrop 8000 spectrophotometer (ThermoScientific) (absorbance ratio A260/280>1.7; A260/230>1.8). DNA integrity was assessed visually by running 25 ng on a 0.8% agarose gel with ethidium bromide. The gel was run at 120 mV for 25 min (see Fig. S1).

### Telomere length measurement

For both the albatross and the zebra finch studies, relative telomere lengths in whole blood were assessed by quantitative real-time PCR amplification (qPCR; Cawthon 2002; Criscuolo et al. 2009). We calculated the ratio of the amount of telomere sequence to the amount of a control gene that is non-variable in copy number in the study species (GAPDH), in comparison to a control sample, producing a single variable for each sample, the T/S ratio. The amount of telomere sequence present in the sample (*T*), and the amount of the control single-copy gene (*S*) are proportional to the number of qPCR amplification cycles needed to reach a threshold fluorescent signal (Ct value) in the exponential phase. The T/S ratio is widely used as a measure of relative telomere length and is suitable for summarizing within-individual changes in telomere length (Nussey et al. 2014); we henceforth refer to it as relative telomere length (RTL). The telomere and GAPDH reactions were carried out on separate plates. For both studies, forward and reverse primers for the GAPDH gene were 5′-AACCAGCCAAGTACGATGACAT-3′ and 5′-CCATCAGCAGCAGCCTTCA-3′ respectively. Telomere primers were: Tel1b (5′-CGGTTTGTTTGGGTTTGGGTTTGGGTTTGGGTTTGGGTT-3′) and Tel2b (5′GGCTTGCCTTACCCTTACCCTTACCCTTACCCTTACCCT-3′). Albatross qPCR analyses for both telomere and GAPDH sequences were performed using 5 ng of DNA with both sets of primers and 1x Absolute blue qPCR SYBR green Low Rox master mix (ThermoFisher Scientific) in a final volume of 25 µl. Primer concentrations in the final mix were 500 nM for the telomere assay and 200 nM for the control gene assay. Zebra finch qPCRs analyses for both telomere sequences and GAPDH were performed using 10 ng of DNA with both sets of primers, in a final volume of 25 µl containing 12.5 µl of 2x Absolute blue qPCR SYBR green Low Rox master mix (ThermoFisher Scientific). Primer concentrations in the final mix were 500 nM for the telomere assay and 70 nM for the GAPDH assay. Real-time amplification of telomere sequences and GAPDH were performed on separate 96-well plates. The telomere thermal profile was 15 min at 95 °C, followed by 27 cycles of 15 s at 95 °C, 30 s at 58 °C, and 30 s at 72 °C. The GAPDH thermal profile was 15 min at 95 °C, followed by 40 cycles of 15 s at 95 °C, 30 s at 60 °C, 30 s, 72 °C. Both assays were followed by melt curve analysis of (58–95 °C 1 °C/5 s ramp).

Each sample (i.e., DNA sample from a given bird stored with either one of the methods) was assayed in triplicate and the mean of the three replicates used. Cross-sectional and longitudinal samples were randomly assigned across plates and repeat samples from the same individual were assayed on the same plate. Each plate also included a calibrator (or golden sample) run in triplicate, which was used to correct for variation among plates, as well as a serial dilution (telomere and GAPDH—40 to 1.25 ng per well) of the calibrator also run in triplicate. Both a negative control (water) and a melting curve were run for each plate to check for specific amplification of a unique amplicon and for the absence of primer-dimer artefacts. For the albatross samples, the average intra-plate variation of the Ct values was 0.62 and 0.40% for the telomere and GAPDH assays, respectively (calculated as the mean of the coefficient of variation of the Ct values for the golden sample on each plate). The average inter-plate variation was 3.02% for the telomere assay and 2.04% for the GAPDH assay (calculated as the coefficient of variation of the Ct values for the golden sample across all plates). For the zebra finch samples, intra-plate mean coefficients of variation for Ct values were 1.4% for the telomere assay and 0.7% for the GAPDH assay. Inter-plate coefficients of variation based on repeated samples were 1.8% for the telomere assay and 1% for the GAPDH assay.

We used the program LinRegPCR to determine the well-specific efficiencies of the PCR reactions and Ct values correcting for background fluorescence (Ruijter et al. 2009). The well-specific efficiencies were used to compare the average reaction efficiency of samples stored in different ways. Relative telomere length for each sample was calculated, following Pfaffl (2001), using average reaction efficiencies for each plate and Ct for each sample determined by LinRegPCR as follows:

$$ {\text{RTL }} = \, \left( {E_{TEL}^{{(CtTEL\left[ {\text{Calibrator}} \right] \, {-} \, CtTEL[{\text{Sample]}})}} } \right)/ \, \left( {E_{GAP}^{{(CtGAP \, \left[ {\text{Calibrator}} \right] \, {-} \, CtGAP \, [{\text{Sample]}})}} } \right) $$

where *E* is the mean reaction efficiency across all samples on a given plate; Ct[Calibrator] is the average Ct across the calibrator triplicate on the plate; and Ct[Sample] is the average Ct across the sample triplicate.

### Statistical analyses

Albatross and zebra finch data were analysed separately using the program R (version 3.2.0). For the albatross samples, relative telomere length (RTL) was log transformed to achieve a normal distribution prior to analysis, though plots show raw RTL for clarity. Significance of terms was assessed using likelihood ratio tests.

Linear mixed effects models (package *lme4*) were used to test whether PCR reaction efficiency varied among samples stored in different ways, for both GAPDH and telomere reactions. We included PCR plate as a fixed multi-level factor to control for among-plate variation in reaction efficiency, and bird ID as a random intercept term to account for repeated measures of individuals. We controlled for the effects of sex and age as they are known to potentially affect telomere length (Hall et al. 2004; Barrett and Richardson 2011).

We then tested for effects of storage treatment method on RTL. For the zebra finch study, we used linear mixed effects models, including bird ID as a random intercept term to account for repeated measures. We included sex (2-level factor), age (linear covariate), and mass (linear covariate) as fixed effects in the model, as well as sample storage method (3-level factor: extracted DNA, frozen whole blood, and FTA cards).

For the albatross samples, the variance in RTL was considerable among sample storage methods (see Results), and so we used generalised least squares (package *nlme*) to include a variance inflation factor for storage method (Zuur et al. 2009). We excluded the limited number of repeat measures of individuals to avoid pseudoreplication, and included fixed effects for sex, age, and sample storage method (as above).

Using only albatross samples collected in 2000 (*n* = 56), we conducted an analysis to see if there was a relationship between RTL and survival over the subsequent 12–14 years (see Supplementary materials). We then used the limited number of longitudinal albatross samples (two measures from 30 individuals) to see if RTL measured in the first sample was correlated with RTL measured 12–14 years later (Pearson’s correlation). Although we may not expect a perfect correlation over this long time period, we may expect the rank order of individuals to remain consistent (Pauliny et al. 2012). Finally, we did pairwise comparisons on the zebra finch data to see if RTL measurements were correlated among the same sample stored in different ways using Pearson’s correlation.

## Results

### Reaction efficiencies

**Fig. 1:**
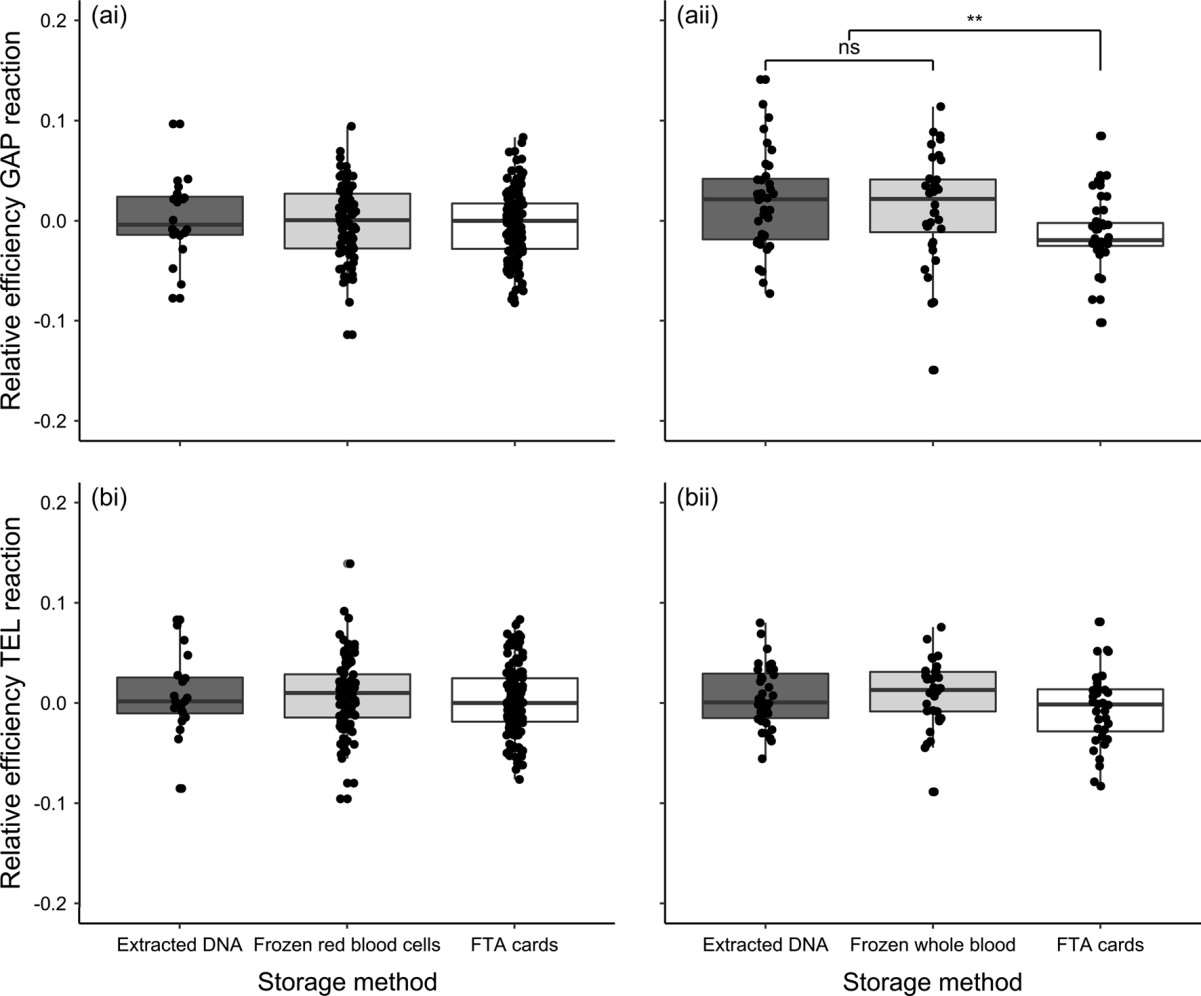
Efficiency of PCR reactions for **i** wandering albatross and **ii** zebra finch samples stored in different ways. Well-specific efficiencies for **a** GAPDH reactions and **b** telomere reactions were estimated using LinRegPCR, and are shown relative to the average for the plate. *Points* represent jittered raw data, with box and whiskers illustrating the median and interquartile range

There was no significant difference in mean PCR reaction efficiency among albatross samples, for either assay, stored in different ways, after accounting for among-plate variation (Fig. 1a; GAPDH: $\chi_{df = 2}^{2}$ = 2.404, *p* = 0.301; telomere: $\chi_{df = 2}^{2}$ = 1.225, *p* = 0.542). For the zebra finch samples, PCR efficiency of GAPDH reactions varied significantly among storage methods ($\chi_{df = 2}^{2}$ = 10.554, *p* = 0.005), but telomere reaction efficiency did not ($\chi_{df = 2}^{2}$ = 4.052, *p* = 0.132). Reaction efficiency of GAPDH reactions was lower for samples stored on FTA cards (Fig. 1b; *β* = −0.031 ± 0.010, *p* = 0.002).

### Factors influencing relative telomere length measurement

#### Albatrosses

**Fig. 2:**
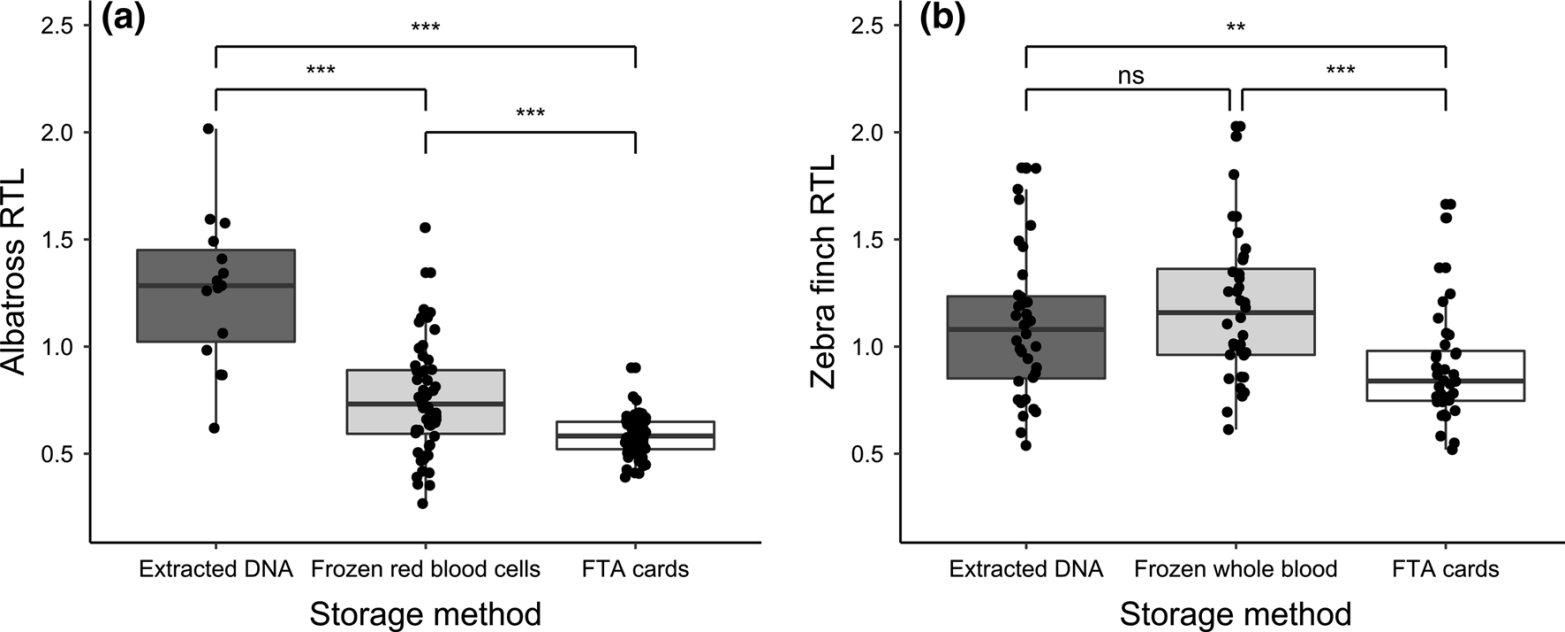
Relative telomere length (RTL) depending on sample storage method in **a** wandering albatrosses and **b** zebra finches. For the albatross study (*n* = 149), the samples were collected from different individuals over a period of years, for different purposes, stored for varying periods of time and extracted in different ways. For the zebra finch study (*n* = 90 from 30 individuals), a single sample was taken from each individual and stored in different ways, keeping other protocols consistent. Points represent jittered raw data, with box and whiskers illustrating the median and interquartile range

**Fig. 3:**
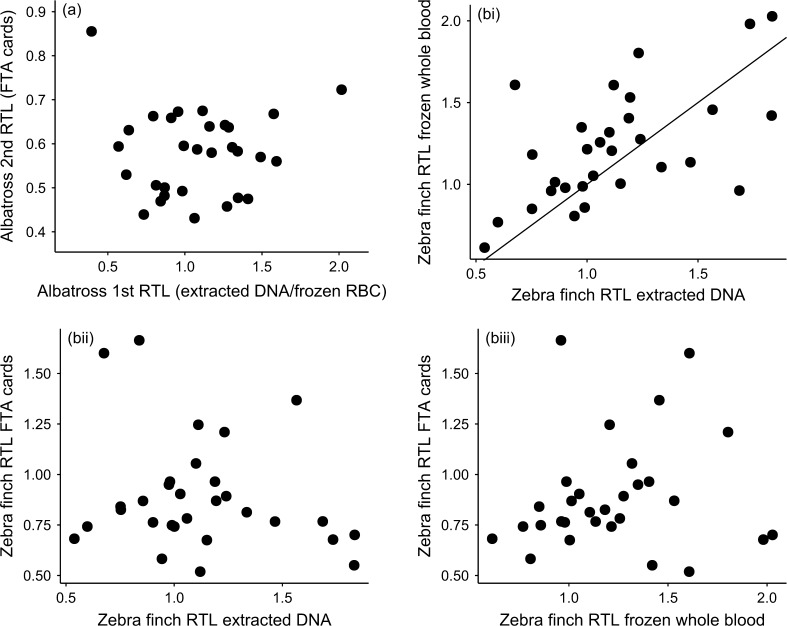
Correlation in RTL measured in samples collected from the same individual wandering albatrosses and zebra finches, but stored in different ways. For the albatross study **a**, samples were collected 12–14 years apart, with the first sample stored either as extracted DNA or as frozen red blood cells (*n* = 30), and the second sample stored on FTA cards. For the zebra finch study **b**, a single sample was collected from each individual (*n* = 30), and this was split and stored in different ways for 2 months. *Points* show raw data. There was a significant correlation between RTL measures in zebra finch samples which were stored as extracted DNA and those that were frozen as whole blood (*r* = 0.593, *p* < 0.001), but none of the other correlations were significant (see Results)

Average relative telomere length (RTL) measured in albatross red blood cells varied depending on sample year and storage method (Fig. 2a; Table 2a). RTL was highest in the samples stored as extracted DNA for over 10 years, intermediate in samples stored as frozen red blood cells over a similar timeframe, and lowest in sample stored on FTA cards for approximately 1 year (Fig. 2a). The variance in RTL also differed significantly among storage methods, being much reduced in samples stored on FTA cards (Fig. 2a; including a variance inflation function significantly improved model fit: $\chi_{df = 2}^{2}$ = 45.431, *p* < 0.001). Males tended to have slightly longer average RTL, but there was no significant relationship between RTL and age, after accounting for sample storage method (Table 2a). In the longitudinal analysis, there was no correlation between RTL measured on the first and second sampling occasions (Fig. 3a; Pearson’s correlation coefficient = −0.008, *p* = 0.686).

**Table 2:** Effect estimates (and standard error) for variables affecting relative telomere length (RTL) measured in samples collected from A wandering albatrosses (*n* = 149) and B zebra finches (*n* = 90 measurements from 30 individuals)

Albatrosses	Estimate	Std error	*P* value
a			
Storage method (frozen red blood cells)	−0.530	0.086	<0.001***
Storage method (FTA cards)	−0.734	0.074	<0.001***
Sex (males)	0.066	0.032	0.042*
Age	−0.001	0.002	0.676

Zebra finches			
b			
Storage method (frozen whole blood)	0.086	0.067	0.198
Storage method (FTA cards)	−0.196	0.067	0.004**
Sex (males)	0.017	0.082	0.833
Age	−0.012	0.019	0.539
Mass	0.009	0.017	0.572

For albatrosses, estimates are from a generalised least squares model including sample storage method as a variance inflation factor. For zebra finches, estimates are from a linear mixed effects model including bird ID as a random effect. The effect of sample storage method is shown relative to samples where long-term storage was as extracted DNA (see Main text for details). Significance of terms was assessed using likelihood ratio tests

Significant values are marked with asterisks

#### Zebra finches

Average RTL measured in zebra finch whole blood samples also varied depending on sample storage method (Fig. 2b; Table 2b). Telomere lengths measured in samples stored for 2 months on FTA cards were significantly shorter than in the same samples stored either as frozen whole blood or as frozen extracted DNA (Fig. 2b; Table 2b). There was no significant relationship between RTL and age, sex, or mass in the zebra finch study (Table 2b). There was a significant correlation between telomere lengths measured in samples that were stored as frozen whole blood for 2 months, and those same samples extracted immediately and stored as extracted DNA (Fig. 3bi; *r* = 0.593, *p* < 0.001). However, RTL measured in samples stored on FTA cards was not correlated with RTL measured in the same samples stored as extracted DNA (Fig. 3bii; *r* = −0.206, *p* = 0.274), or as frozen whole blood (Fig. 3biii; *r* = 0.133, *p* = 0.483). As a check for accuracy, we re-ran a random subset (*n* = 23, 7 fresh blood samples, 8 FTA cards samples and 8 frozen blood samples) of the zebra finch samples and found an estimated reliability of 0.90—intra-class correlation (ICC fresh samples = 0.99; ICC frozen samples = 0.92; ICC FTA cards samples = 0.8) and a good correlation between the runs (*r* = 0.91, Pearson correlation; fresh samples *r* = 0.99, frozen samples *r* = 0.92, FTA cards samples *r* = 0.8).

## Discussion

In this study, we find evidence that sample storage method influences telomere length measurement by qPCR in birds. In our wandering albatross study, we used archived samples from this population collected over a period of 14 years, which were subject to different treatment and handling protocols. We find that mean relative telomere length (RTL) differs significantly among samples stored in three different ways; longest among samples subject to long-term storage as extracted DNA, intermediate in samples stored as frozen red blood cells, and shortest among samples stored on FTA cards (Fig. 2a). The range of telomere lengths also varied between groups, with a reduction in variance among samples stored on FTA cards. However, the sample year, duration of storage, and DNA extraction method also varied among samples, and these effects are confounded with storage method. We, therefore, designed our zebra finch experiment to test specifically for impacts of different storage methods on RTL measurement in a controlled manner, taking a single sample from 30 individuals and storing them in three different ways while keeping all other protocols identical. Again, we find that RTL measurement differs significantly depending on storage method; RTL of samples stored on FTA cards for 2 months was significantly shorter than in samples extracted immediately or as frozen whole blood for the same period (Fig. 2b). More importantly, there was no correlation between RTL measures from the same samples stored on FTA cards *vs.* samples stored in other ways (Fig. 3b). This study highlights the importance of consistency of sampling protocol in telomere length studies, particularly in the context of long-term field studies. It also suggests that FTA cards should only be used as a long-term storage solution after validation of telomere length measurement in a particular study.

The wandering albatross samples used in this study were collected for different purposes over a period of years, and, therefore, were not treated in a consistent fashion (Table 1a). This may be a common feature of long-term field studies such as the albatrosses on Bird Island, as researchers addressing a particular question often try to capitalise on archived samples as potentially valuable resources. We hoped to combine the detailed life-history data available for this study population with information on telomere lengths to provide insights into age-related variation in this very long-lived species (Froy et al. 2013). However, the confounding effects of sample treatment and storage mean that we can gain a little biological insight about the study species from this study. The only insight that we were able to gain was from the comparison of survival rates of individuals that were sampled in the same year, for which sampling protocol was consistent (see Supplementary material). Mean telomere length measured in albatross samples stored as extracted DNA for over 10 years was longer than in those stored as frozen red blood cells over a similar time period (Fig. 2a). This could be because extracted DNA is more stable than whole red blood cells over the long term (Madisen et al. 1987). However, these samples were also subject to different methods of DNA extraction, with the red blood cell samples extracted using a spin-column based method as opposed to the salting-out method used for the earlier samples (Table 1a). It has been suggested that spin-column based methods of DNA extraction could result in shorter telomere length measurements due to shearing of the DNA (Cunningham et al. 2013). We, therefore, think that it is likely that the extraction methodology accounts for the observed differences in RTL between samples of different provenance, rather than the storage method. This assertion is supported by the lack of observed difference in RTL measured in zebra finch samples stored either as extracted DNA or as frozen whole blood (Fig. 2b). Furthermore, RTL measured in albatross samples stored on FTA cards for 1 year was significantly shorter than those in samples stored as frozen red blood cells (Fig. 2a). However, these samples were both extracted using spin-column based methods (Table 1a). We also observe a collapse in the variance of RTL in samples stored on FTA cards (Fig. 2a), which is cause for concern. There was no significant difference in mean reaction efficiency among albatross samples stored in different ways (Fig. 1a). After the telomere assays had been run, ~25% of samples stored as extracted DNA or frozen red blood cells were run on gels and DNA integrity was acceptable. Eight samples stored on FTA cards were also run, but these showed signs of degradation. However, it was not clear when this degradation occurred. Since DNA degradation has been shown to influence RTL measurement in some cases (Tolios et al. 2015), our results suggest that, perhaps, the FTA samples were degraded.

In our follow-up study on zebra finches, telomere length also appeared shorter in samples stored on FTA cards than those stored as extracted DNA or frozen whole blood (Fig. 2b). In this study, unlike the albatross study, the same sample was divided and stored in three different ways, and so we expected a very high correlation among RTL measures. Results from samples stored as extracted DNA or frozen whole blood were, indeed, correlated (Fig. 3bi), but there was no significant correlation between samples stored on FTA cards and either alternative storage method (Fig. 3bii, biii). This could be due to degradation of the FTA samples, since the DNA integrity gels that we ran on the zebra finch samples suggest some DNA fragmentation (Fig. S1). We also found that mean PCR reaction efficiency was significantly lower for the GAPDH reaction for FTA card samples (Fig. 1b). This could again be due to sample degradation, if this interferes with primer binding, or, perhaps, could suggest some kind of contamination with PCR inhibitors. However, the efficiency of the telomere reactions did not differ significantly among storage methods (Fig. 1b). This difference between the efficiency of GAPDH and telomere reactions will have consequences for RTL, with lower reaction efficiency leading to an underestimate of the amount of the control gene, and, therefore, an overestimate of the relative amount of telomeric DNA and an overestimate in RTL. However, this does not explain our findings, since we find shorter, rather than longer telomeres in FTA samples (Fig. 2b). Our findings for FTA cards contrast with the only other study of which we are aware that tested for impacts of FTA card storage on telomere length measurement; Zanet et al. (2013) found that telomere length in human blood samples stored on FTA cards was highly correlated with that in fresh and frozen whole blood samples, and that mean RTL was higher for FTA samples. However, that study focused on leukocyte telomere length in humans, rather than red blood cell telomere length in birds. It also used a much shorter storage period (9 days) than our study, and also slightly different cards (Whatman 903 Multipart Neonatal cards), which may mean that their samples were better preserved and could help to explain these inconsistencies.

To conclude, we find that storage method may have consequences for measurements of telomere length, and in particular that measurements from samples stored on FTA cards may not be equivalent to samples stored as extracted DNA or as frozen whole blood over long periods. This supplements the growing body of evidence that suggests that careful consideration of methodology (collection, storage, and extraction) is essential when measuring telomere length by qPCR, which is particularly important for long-term field studies. It also suggests that further validation is required before using FTA cards as a long-term storage solution for blood samples for telomere length analysis. Our results emphasise that consistency in sampling protocol is very important, unless equivalence between methods is clearly demonstrated, since confounding effects may otherwise obscure potential biological inferences.

## Electronic supplementary material

Below is the link to the electronic supplementary material.

Supplementary material 1 (DOCX 943 kb)

## References

[CR1] Armanios M, Blackburn EH (2012). The telomere syndromes. Nat Rev Genet.

[CR2] Aubert G, Lansdorp PM (2008). Telomeres and aging. Physiol Rev.

[CR3] Aviv A, Hunt SC, Lin J, Cao X, Kimura M, Blackburn E (2011). Impartial comparative analysis of measurement of leukocyte telomere length/DNA content by Southern blots and qPCR. Nucleic acids Res.

[CR4] Barrett ELB, Richardson DS (2011). Sex differences in telomeres and lifespan. Aging Cell.

[CR5] Bize P, Criscuolo F, Metcalfe NB, Nasir L, Monaghan P (2009). Telomere dynamics rather than age predict life expectancy in the wild. Proc R Soc Lond B.

[CR6] Blackburn EH (1991). Structure and function of telomeres. Nature.

[CR7] Blackburn EH (2000). Telomere states and cell fates. Nature.

[CR8] Burg TM, Croxall JP (2001). Global relationships amongst black-browed and grey-headed albatrosses: analysis of population structure using mitochondrial DNA and microsatellites. Mol Ecol.

[CR9] Cawthon RM (2002). Telomere measurement by quantitative PCR. Nucleic Acids Res.

[CR1001] Criscuolo F, Bize P, Nasir L (2009). Real-time quantitative PCR assay for measurement of avian telomeres. J Avian Biol.

[CR10] Croxall JP, Rothery P, Pickering SPC, Prince PA (1990). Reproductive performance, recruitment and survival of wandering albatrosses Diomedea exulans at Bird Island, South Georgia. J Anim Ecol.

[CR11] Cunningham JM (2013). Telomere length varies by DNA extraction method: implications for epidemiologic research. Cancer Epidemiol Prev Biomark.

[CR12] Denham J, Marques FZ, Charchar FJ (2014). Leukocyte telomere length variation due to DNA extraction method. BMC Res Notes.

[CR13] Froy H, Phillips RA, Wood AG, Nussey DH, Lewis S (2013). Age-related variation in reproductive traits in the wandering albatross: evidence for terminal improvement following senescence. Ecol Lett.

[CR14] Hall ME (2004). Telomere loss in relation to age and early environment in long-lived birds. Proc R Soc Lond B.

[CR15] Harley CB, Futcher AB, Greider CW (1990). Telomeres shorten during ageing of human fibroblasts. Nature.

[CR16] Haussmann MF, Salomons HM, Verhulst S (2011). Telomere measurement tools: telometric produces biased estimates of telomere length. Heredity-Basingstoke.

[CR17] Heidinger BJ, Blount JD, Boner W, Griffiths K, Metcalfe NB, Monaghan P (2012). Telomere length in early life predicts lifespan. Proc Natl Acad Sci.

[CR18] Hofmann JN (2014). Telomere length varies by DNA extraction method: implications for epidemiologic research—letter. Cancer Epidemiol Prev Biomark.

[CR19] Horn T, Robertson BC, Gemmell NJ (2010). The use of telomere length in ecology and evolutionary biology. Heredity.

[CR20] Lin J, Epel E, Blackburn E (2012). Telomeres and lifestyle factors: roles in cellular aging. Mutat Res.

[CR21] Madisen L, Hoar DI, Holroyd CD, Crisp M, Hodes ME, Reynolds JF (1987). The effects of storage of blood and isolated DNA on the integrity of DNA. Am J Med Genet.

[CR22] Martin-Ruiz CM (2014). Reproducibility of telomere length assessment: an international collaborative study. Int J Epidemiol.

[CR23] Monaghan P (2010). Telomeres and life histories: the long and the short of it. Ann N Y Acad Sci.

[CR24] Monaghan P, Haussmann MF (2006). Do telomere dynamics link lifestyle and lifespan?. Trends Ecol Evol.

[CR25] Montpetit AJ (2014). Telomere length: a review of methods for measurement. Nurs Res.

[CR26] Nakagawa S, Gemmell NJ, Burke T (2004). Measuring vertebrate telomeres: applications and limitations. Mol Ecol.

[CR27] Nussey DH (2014). Measuring telomere length and telomere dynamics in evolutionary biology and ecology. Methods Ecol Evol.

[CR28] Olsen MT, Bérubé M, Robbins J, Palsbøll PJ (2012). Empirical evaluation of humpback whale telomere length estimates; quality control and factors causing variability in the singleplex and multiplex qPCR methods. BMC Genet.

[CR29] Pauliny A, Larsson K, Blomqvist D (2012). Telomere dynamics in a long-lived bird, the barnacle goose. BMC Evol Biol.

[CR30] Pezzoli N (2007). Quantification of mixed chimerism by real time PCR on whole blood-impregnated FTA cards. Leuk Res.

[CR1002] Pfaffl MW (2001). A new mathematical model for relative quantification in real-time RT-PCR. Nucleic Acid Res.

[CR31] Raschenberger J (2016). Influence of DNA extraction methods on relative telomere length measurements and its impact on epidemiological studies. Sci Rep.

[CR32] Ruijter JM (2009). Amplification efficiency: linking baseline and bias in the analysis of quantitative PCR data. Nucleic Acids Res.

[CR33] Seeker LA (2016). Method specific calibration corrects for DNA extraction method effects on relative telomere length measurements by quantitative PCR. PLoS ONE.

[CR34] Sharma A, Jaiswal S, Shukla M, Lal J (2014). Dried blood spots: concepts, present status, and future perspectives in bioanalysis. Drug Test Anal.

[CR35] Smith S, Turbill C, Penn DJ (2011). Chasing telomeres, not red herrings, in evolutionary ecology. Heredity.

[CR36] Stier A, Reichert S, Criscuolo F, Bize P (2015). Red blood cells open promising avenues for longitudinal studies of ageing in laboratory, non-model and wild animals. Exp Gerontol.

[CR37] Tolios A, Teupser D, Holdt LM (2015). Preanalytical conditions and DNA isolation methods affect telomere length quantification in whole blood. PLoS ONE.

[CR38] von Zglinicki T (2002). Oxidative stress shortens telomeres. Trends Biochem Sci.

[CR39] Walsh PS, Metzger DA, Higuchi R (1991). Chelex 100 as a medium for simple extraction of DNA for PCR-based typing from forensic material. Biotechniques.

[CR40] Zanet DL, Saberi S, Oliveira L, Sattha B, Gadawski I, Côté HCF (2013). Blood and dried blood spot telomere length measurement by qPCR: assay considerations. PLoS ONE.

[CR41] Zuur A, Ieno EN, Meesters E (2009). A beginner’s guide to R.

