# Supplementary material for: Telomere length measurement by qPCR in birds is affected by storage method of blood samples
Source: Oecologia. 2017 May 25;184(2):341–50. doi: 10.1007/s00442-017-3887-3 (PMC5487852; doi:10.1007/s00442-017-3887-3)

Supplementary materials

Figure S1. Example of DNA integrity gel for two zebra finch samples stored as extracted DNA (F), frozen whole blood (Fr), whole blood on FTA cards (FTA).


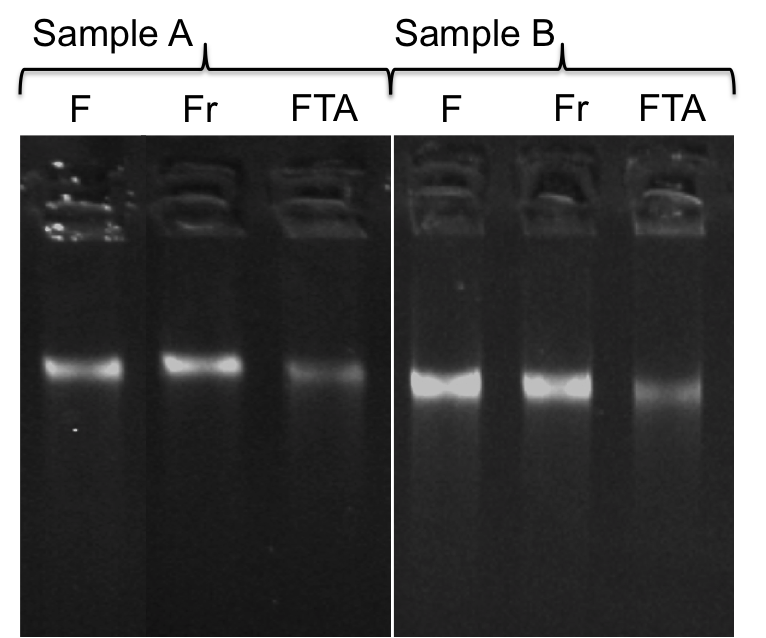


**Telomere length does not predict subsequent survival in the wandering albatross**

We examined the relationship between RTL and subsequent survival over a 12 year period using a subset of data which was not affected by differences in sample storage or treatment. For albatross samples collected in 2000 and stored as frozen red blood cells (n = 56), we used a Cox proportional hazards model (package *survival*) to examine the relationship between initial telomere length and survival. We used 2008 as the censor point to be certain that individuals who were not observed were not just skipping breeding. -Survival was modelled as a function of RTL while accounting for the effects of sex (2-level factor) and age (linear covariate).

There was no association between RTL measured in 2000 and survival over the subsequent 12 years (Fig. S2; β = -0.531 ± 0.588, p = 0.544). We did not detect an effect of sex on survival (β = -0.400 ± 0.670, p = 0.346), but likelihood of death was related to age, with birds that were older on first sampling less likely to survive the follow-up period (β = 0.091 ± 0.036, p = 0.011).

Figure S2. Relative telomere length of wandering albatrosses measured in 2000 (n = 56) does not predict subsequent survival.


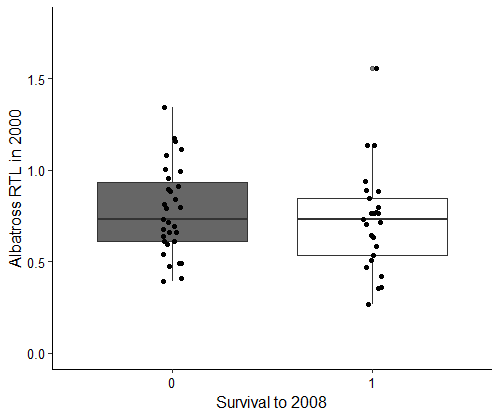

Supplement: Supplementary file 1 — Supplementary material 1 (DOCX 943 kb) [file 442_2017_3887_MOESM1_ESM.docx]
